# Mechanistic insights into super-enhancer-related genes as prognostic signatures in colon cancer

**DOI:** 10.18632/aging.205906

**Published:** 2024-06-07

**Authors:** Yini Tang, Shuliu Sang, Shuang Gao, Weina Xu, Hailun Zhou, Xiaoting Xia

**Affiliations:** 1 Department of Endoscopy, Shuguang Hospital Affiliated to Shanghai University of Traditional Chinese Medicine, Shanghai, China; 2 Department of Oncology, Yueyang Hospital of Integrated Traditional Chinese and Western Medicine, Shanghai University of Traditional Chinese Medicine, Shanghai, China; 3 Department of Anorectal Surgery, The Third Affiliated Hospital of Yunnan University of Traditional Chinese Medicine, Yunnan, China; 4 Department of TCM, Zhoujiadu Community Health Service of Shanghai Pudong New Area Center, Shanghai, China; 5 Department of Oncology, Shanghai TCM-intergrated Hospital, Shanghai, China

**Keywords:** colon cancer, prognosis, super enhancer, immune infiltration, LZTS2

## Abstract

Background: Colon cancer (CC) is the most frequently occurring digestive system malignancy and is associated with a dismal prognosis. While super-enhancer (SE) genes have been identified as prognostic markers in several cancers, their potential as practical prognostic markers for CC patients remains unexplored.

Methods: We obtained super-enhancer-related genes (SERGs) from the Human Super-Enhancer Database (SEdb). Transcriptome and relevant clinical data for colon cancer (CC) were sourced from the Gene Expression Omnibus (GEO) database. Subsequently, we identified up-regulated SERGs by the Weighted Gene Co-expression Network Analysis (WGCNA). Prognostic signatures were constructed via univariate and multivariate Cox regression analysis. We then delved into the mechanisms of these predictive genes by examining immune infiltration. We also assessed differential sensitivities to chemotherapeutic drugs between high- and low-SERGs risk patients. The critical gene was further validated using external datasets and finally confirmed by qRT PCR.

Results: We established a ten-gene risk score prognostic model (S100A11, LZTS2, CYP2S1, ZNF552, PSMG1, GJC1, NXN, and DCBLD2), which can effectively predict patient survival rates. This model demonstrated effective prediction capabilities in survival rates at 1, 3, and 5 years and was successfully validated using external datasets. Furthermore, we detected significant differences in immune cell infiltration between high- and low-SERGs risk groups. Notably, high-risk patients exhibited heightened sensitivity to four chemotherapeutic agents, suggesting potential benefits for precision therapy in CC patients. Finally, qRT-PCR validation revealed a significant upregulation of LZTS2 mRNA expression in CC cells.

Conclusion: These findings reveal that the SERGs model could effectively predict the prognosis of CC.

## INTRODUCTION

Colon cancer (CC) is the leading cause of cancer-related mortality worldwide, and it’s one of the common digestive system tumors [1]. Treatment options for CC include surgical tumor removal, chemotherapy, radiation therapy, and others [2]. The choice of treatment options depends on the stage and location of the tumor and the overall condition of the patient. However, the 5-year survival rate of advanced high-grade CC patients is less than 10% [3]. It is crucial to effectively determine the prognosis of CC patients as well as to provide rational treatment options. Therefore, our objective was to create a prognostic model functioning as the prognostic marker for CC.

Super-enhancers (SEs), an exceptional group of cis-regulatory elements, are characterized by their aggregation of multiple neighboring enhancers [4, 5]. They have been recognized as crucial oncogenic drivers for preserving the identity of cancer cells [6]. Aberrant SEs frequently assemble to activate proto-oncogenes or other genes vital for cancer cells, instigating tumorigenesis, promoting tumor proliferation, and enhancing the adaptability of cancer cells within the tumor microenvironment [7]. Suppression of the cellular machinery necessary for super-enhancer (SE) assembly and upkeep hinders oncogenic transcription, consequently impeding tumor growth [8]. More and more pieces of evidence show that SEs are effective biomarkers in cancer [9–11]. However, SEs as effective prognostic markers for CC patients remain unassessed. Consequently, it is crucial to investigate the possible molecular mechanisms and prognostic markers associated with CC by focusing on SEs.

In this study, the GSE39582 and the Human Super Enhancer Database (SEdb) database [12] were utilized to analyze SE-related genes (SERGs) in CC and develop the prognostic model. Subsequently, we employed the TCGA-COAD dataset to validate the predictive efficacy of the model. Additionally, we delved into the correlation between the risk model and immune in CC. Moreover, we predicted chemotherapeutic drug sensitivity and prognosis for CC patients. These findings have the potential to differentiate high-risk patients, enhancing the opportunity for personalized therapy and thereby improving patient survival rates.

## METHODS

### Data source

The GSE39582 dataset from the Gene Expression Omnibus (GEO, https://www.ncbi.nlm.nih.gov/geo/) database was defined as the training set for COAD, which contains 566 patients with COAD and 19 control population. The TCGA (Cancer Genome Atlas database, http://cancergenome.nih.gov) database was defined as the testing set for 467 COAD tissues and 41 normal tissues. The SERGs were derived from the SEdb database for HCT116 and HT29 cell lines and are shown in Supplementary Table 1.

### Construction of WGCNA

Weighted Gene Co-expression Network Analysis (WGCNA) is performed as a systematic biology algorithm designed to construct gene co-expression networks and elucidate gene correlations across multiple dimensions. In this study, we utilized the R package “WGCNA” to estimate the COAD modules of correlated genes. Before the analysis, outliers were filtered out using the cutreeStatic function found within WGCNA. Subsequently, with a soft threshold power of 6 (b = 6), we generated the adjacency matrix to optimally fit the network structure. Pearson correlations were calculated between gene expression levels to construct the correlation matrix of genes, which established the connectivity between the nodes. Utilizing a hierarchical clustering dendrogram of this matrix, we built a topological overlap matrix to segregate different modules that follow similar gene expression patterns. The module eigengene (ME) expression profiles were then identified by amalgamating the expression profiles of each module, aiming to uncover a link between ME and clinical status. Candidate modules demonstrating significant correlation coefficients with clinical traits were subsequently shortlisted.

### Enrichment analysis of SERGs

The genes from positive correlation coefficient modules of COAD in WGCNA and SERGs were intersected and visualized by using the Jvenn. Then, we analyzed functional annotations of these shared genes using GO and KEGG enrichment analysis.

### Establish a prognostic risk model for SERGs

Univariate and multivariate Cox analysis, as the important means of determining prognostic-related SERGs in COAD, were performed for identification. Then, using the prognostic-related prediction formulas obtained by multivariate Cox regression analysis, the prognostic model was constructed in the R survival package. Afterwards, the high- and low-risk groups were differentiated by Kaplan Meier (K-M) survival analysis. Subsequently, the predictive value of the prognostic model was evaluated by the receiver operating characteristic area (ROC).

### Immune profile analysis

We conducted a series of analyses to identify the differences between high- and low-SERGs risk groups in immune-cell infiltration. The abundance of 28 immune-cell types was determined using the ssGSEA. The Wilcoxon rank-sum test was applied to evaluate differences in immune cell proportions.

### Single-cell analysis

We obtained single-cell RNA sequencing (scRNA-seq) data of COAD patients from the GEO database numbered GSE200997 and performed analysis using the ‘Seurat’ package. Subsequently, we identified immune cell clusters using the ‘FindNeighbors’ and ‘FindClusters’ functions. To evaluate the expression of key genes within the same type of immune cells between the tumor and normal groups, we utilized the ‘FindAllMarkers’ function.

### Chemotherapeutic sensitivity

To predict the sensitivity of chemotherapeutic drugs between high- and low-SERGs risk groups, we utilized the pRRophetic R package to project drug sensitivity values (IC_50_) by constructing a ridge regression model [13]. We selected several common anticancer drugs for this analysis, including camptothecin, docetaxel, gefitinib, gemcitabine, pazopanib, and sunitinib.

### Hub genes validation in CC

To confirm the hub gene in CC, we performed the expression of ten genes on GSE39582 and TCGA-COAD via the Wilcoxon rank-sum test and ggplot2 with cutoff *P*-value < 0.05, respectively. The intersection of hub genes in the GSE39582 and TCGA-COAD was identified. The hub genes were identified using the GEPIA2 database based on “overall survival,” with the cutoff values determined using the “median” group. *P* < 0.05, *P* (HR) < 0.05 were significant. Subsequently, we acquired immunohistochemistry (IHC) images of CC and normal tissues via the Human Protein Atlas (HPA) portal [14].

### Cell culture

CC cell lines (HCT116 and HT29 cells) and NCM460 cells were acquired from the Cell Bank of the Chinese Academy of Sciences. HCT116, HT29, and NCM460 cells were grown in the DMEM medium (10%-FBS) in a 37°C incubator with 5% CO_2_.

### qRT-PCR validation of the key gene

We isolated the total RNA from cells via TRIzol reagent (Invitrogen, USA). For cDNA synthesis, a cDNA Synthesis kit (Invitrogen, Thermo Fisher Scientific Inc., USA) was performed to reverse the transcription reaction into cDNA. The relative mRNA levels were assessed by the 2^−ΔΔCq^ calculation method and normalized by GAPDH mRNA expression. Primers were as follows: LZST2 forward primers: GGTGGCCCTATGACTTGG, reverse primers: AGCGGTGGGGAATGAAG. S100A11 forward primers: ATGGCAAAAATCTCCAGCCCT, reverse primers: TGTGAAGGCAGCTAGTTCTGTA. CYP2S1 forward primers: GCGCTGTATTCAGGGCTCAT, reverse primers: CTTCCAGCATCGCTACGGTT. PSMG1 forward primers: TCCTTTCCTGAGAGCCCTAAAA, reverse primers: TGTTCTAGCAATGGACAACACG. DCBLD2 forward primers: ATGTGGACACACTGTACTAGGC, reverse primers: CTGTTGGGATAGGTCTGTGGG. GAPDH forward primers: GGAAGCTTGTCATCAATGGAAATC, reverse primers: TGATGACCCTTTTGGCTCCC.

### Western blot assay

CC cell lines (HCT116 and HT29 cells) and NCM460 cells were lysed in ice-cold RIPA lysis buffer (Beyotime Inc., China, P0013B). The quantification of protein content was achieved by utilizing the BCA Protein Assay Kit (Epizyme Biotech, China, ZJ101). The proteins were resolved via SDS-PAGE and subsequently transferred onto PVDF membranes. To block non-specific binding, the membranes were incubated with a solution containing 5% milk. Subsequently, the membranes were incubated with the corresponding primary antibodies overnight at 4°C. The horseradish Peroxidase secondary antibodies were applied and detected using an ECL solution (New Cell Molecular Biotech, China, P10200). Primary antibodies against LZTS2 (15677-1-AP) and GAPDH (60004-1-Ig) were sourced from Proteintech.

### Statistical analysis

Statistical analyses were conducted using R (version 4.3.1) and GraphPad Prism. The *T*-test was performed to reveal the statistical differences between the two groups and One-way ANOVA analysis was for comparisons involving multiple groups. Statistical significance was determined at *P* < 0.05.

### Data availability statement

The raw data are encompassed within the article and supplementary material.

## RESULTS

### Identification of SERGs

366 and 859 SERGs were identified in HCT116 and HT29 cell lines from the SEdb database, respectively. After removing the duplicates, a total of 974 SERGs were obtained.

### Co-expression modules in LUAD and PAH

**Figure 1:**
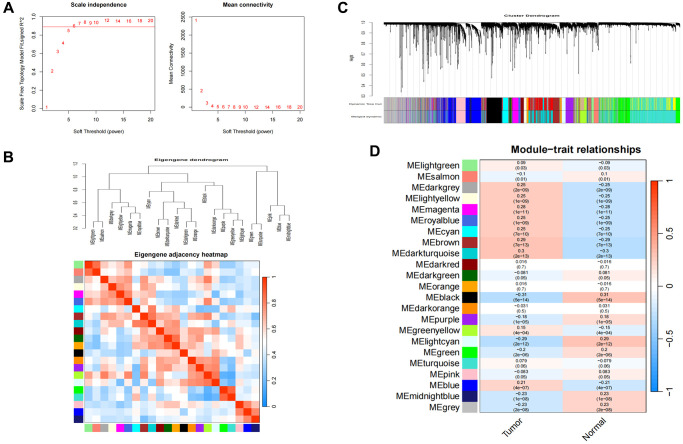
**Consensus module analysis of CC using WGCNA.** (**A**) Scale-free topology model fit (R^2^ > 0.98) and mean connectivity. (**B**) Association between the gene modules. (**C**) Cluster dendrogram for CC. (**D**) Heatmap analysis of modules and clinical features in CC.

To identify the module genes associated with the disease, 23 modules were generated in GSE39582 by WGCNA, and different colors represented different modules. Then, we mapped the heat map, which could assess the association between modules and the disease based on the Spearman correlation coefficient (Figure 1). Considering the association of SEs with gene expression in tumors, most of the related genes may probably function as oncogenes [15]. Given that SEs enrich transcription factors to enhance gene expression, we specifically selected the modules “lightgreen”, “darkgrey”, “lightyellow”, “magenta”, “royablue”, “cyan”, “brown”, “darkturquoise”, “greenyellow”, and “blue” as COAD-related modules, which were positively correlated with COAD (lightgreen: r = 0.09, *p* = 0.03, genes = 209; darkgrey module: r = 0.25, *p* = 2e−09, genes = 147; lightyellow module: r = 0.25, *p* = 1e−09, genes = 203; magenta module: r = 0.28, *p* = 1e−11, genes = 654; royablue module: r = 0.25, *p* = 1e−09, genes = 191; cyan module: r = 0.25, *p* = 7e−10, genes = 398, brown module: r = 0.29, *p* = 7e−13, genes = 1612, darkturquoise module: r = 0.3, *p* = 2e−13, genes = 1770, greenyellow module: r = 0.15, *p* = 4e−04, genes = 996, blue module: r = 0.21, *p* = 4e−07, genes = 169).

### Enrichment analyses of shared genes

**Figure 2:**
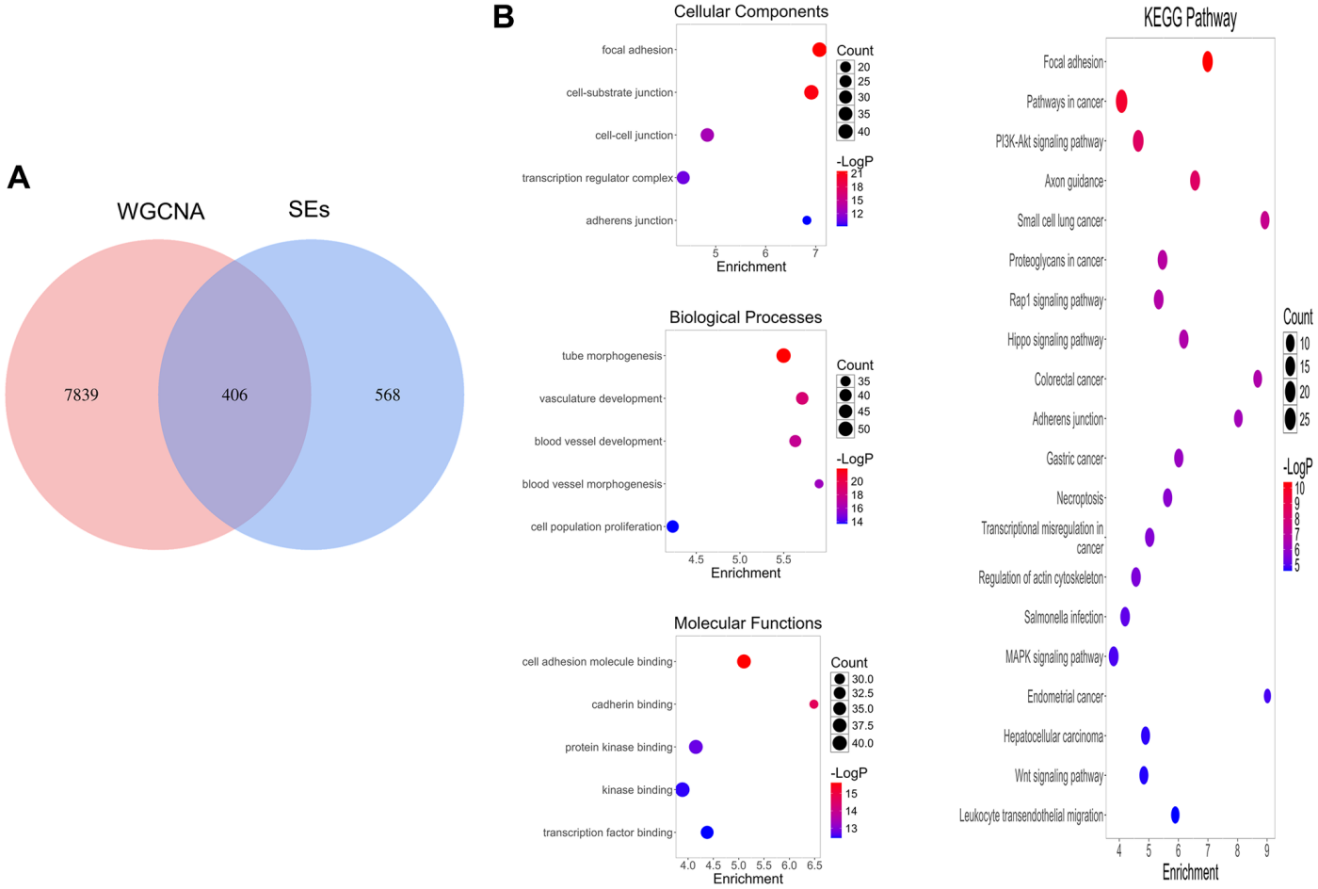
**SERGs screening and function enrichment analysis.** (**A**) Identification of pivotal SERGs in COAD. (**B**) GO and KEGG analysis of SERGs.

A total of 406 genes at the intersection of WGCNA positive-correlated modules and SERGs were considered to be connected with the pathogenesis of COAD (Figure 2A). GO and KEGG analyses were utilized to identify 406 genes’ biological functions and essential pathways. The results indicated that the biological process (BP) was mainly enriched in tube morphogenesis and vasculature development, etc. The cellular component (CC) was primarily enriched in focal adhesion, etc. The molecular function (MF) was especially involved in cell adhesion molecule binding, etc., and KEGG analysis was primarily related to Pathways in cancer and PI3K-Akt signaling pathway (Figure 2B).

### Establishment of the SERGs prognostic model

**Figure 3:**
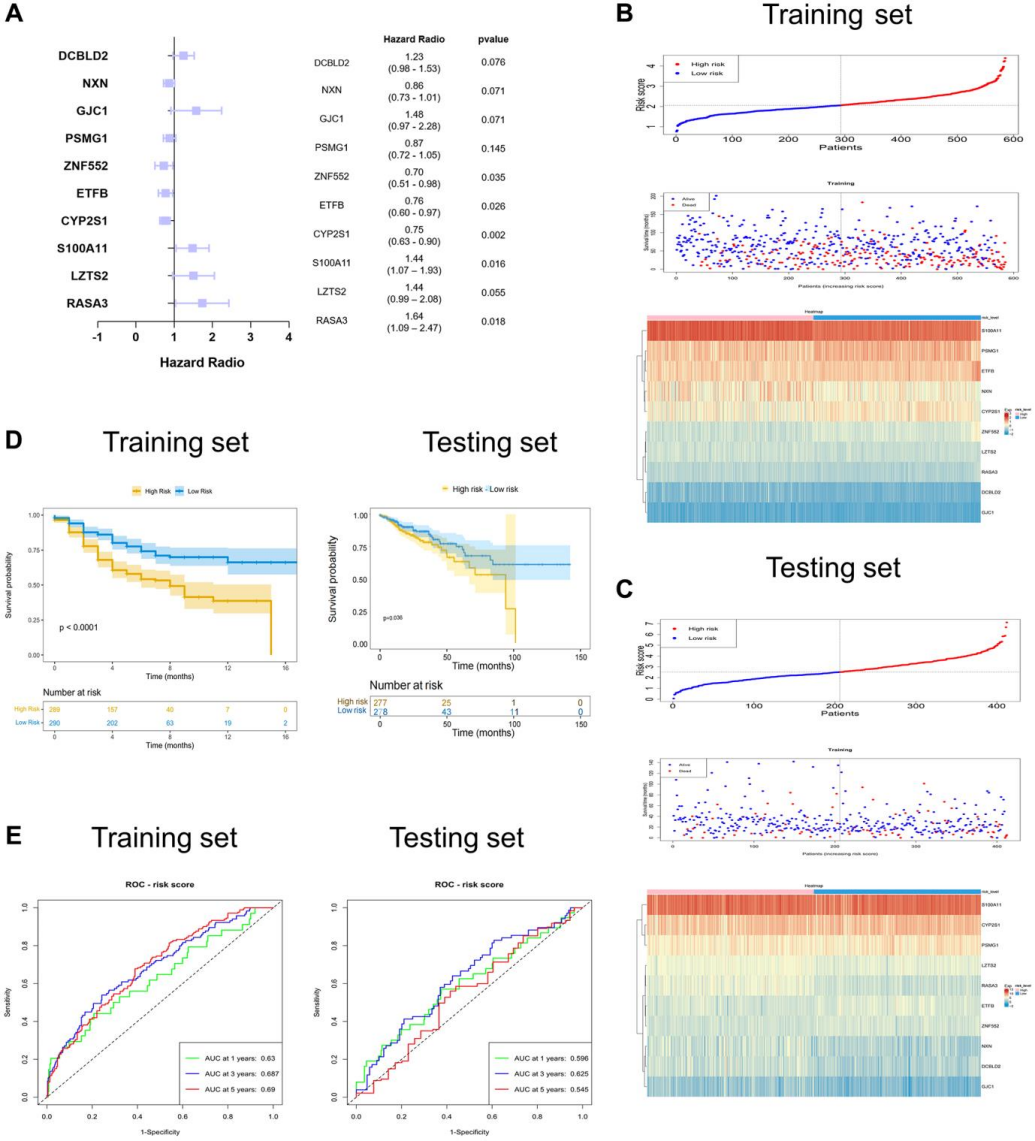
**Identification and validation of the ten-SERGs risk model.** (**A**) The forest plot of ten-SERGs prognostic model. The risk score distribution, survival status, and heat map of ten SERGs in (**B**) training set and (**C**) testing set. (**D**) Patients in low-risk groups had longer OS in the training set and testing set. (**E**) The ROC analysis of the SERGs risk model in the training set and testing set.

To further assess the SERGs associated with the prognosis of COAD and construct the model, we initially conducted univariate Cox analysis, considering genes with *P* < 0.01 (Supplementary Table 2). The *P*-value of the model (S100A11, LZTS2, RASA3, CYP2S1, ETFB, ZNF552, PSMG1, GJC1, NXN, and DCBLD2) was 2.3608e-09, with an AIC of 2225.98 by multivariate Cox analysis (Figure 3A). Even though the *P*-values for LZTS2, PSMG1, GJC1, NXN, and DCBLD2 exceed 0.05, it’s noteworthy that the model still exhibits the lowest AIC value among the tested models. We then proceeded to create a ten-gene SERGs model, which was formulated using the expression levels of each gene and their respective coefficients: risk score = (0.362746416 × S100A11) + (0.361912228 × LZTS2) + (0.495106924 × RASA3) + (−0.284872577 × CYP2S1) + (−0.274930212 × ETFB) + (−0.350131731 × ZNF552) + (−0.138081346 × PSMG1) + (0.395225421 × GJC1) + (−0.153361139 × NXN) + (0.203044412 × DCBLD2). Among the seven genes (S100A11, LZTS2, RASA3, GJC1 and DCBLD2) were classified as risk-related genes (HR > 1), while CYP2S1, ETFB, ZNF552, PSMG1 and NXN were protective genes (HR < 1). The risk score classified patients into high- and low-risk groups using the median as the threshold. To validate the signature of SERGs, we calculated the risk scores of patients in the TCGA-COAD dataset. The survival status in the two groups is shown in Figure 3B and gene expression in Figure 3C. Patients in the high-risk group suffered the worse OS. The validation results were largely consistent with those obtained from the GSE39582 dataset: the high-risk group endured worst OS (Figure 3D). Additionally, the AUCs at 1, 3, and 5 years for OS were 0.631, 0.684, and 0.681 in the GSE39582 dataset, respectively. The AUCs for OS at 1, 3, and 5 years were found to be 0.681, 0.702, and 0.633 in the TCGA-COAD dataset, respectively (Figure 3E).

### Immune cell infiltration with SERGs risk group

**Figure 4:**
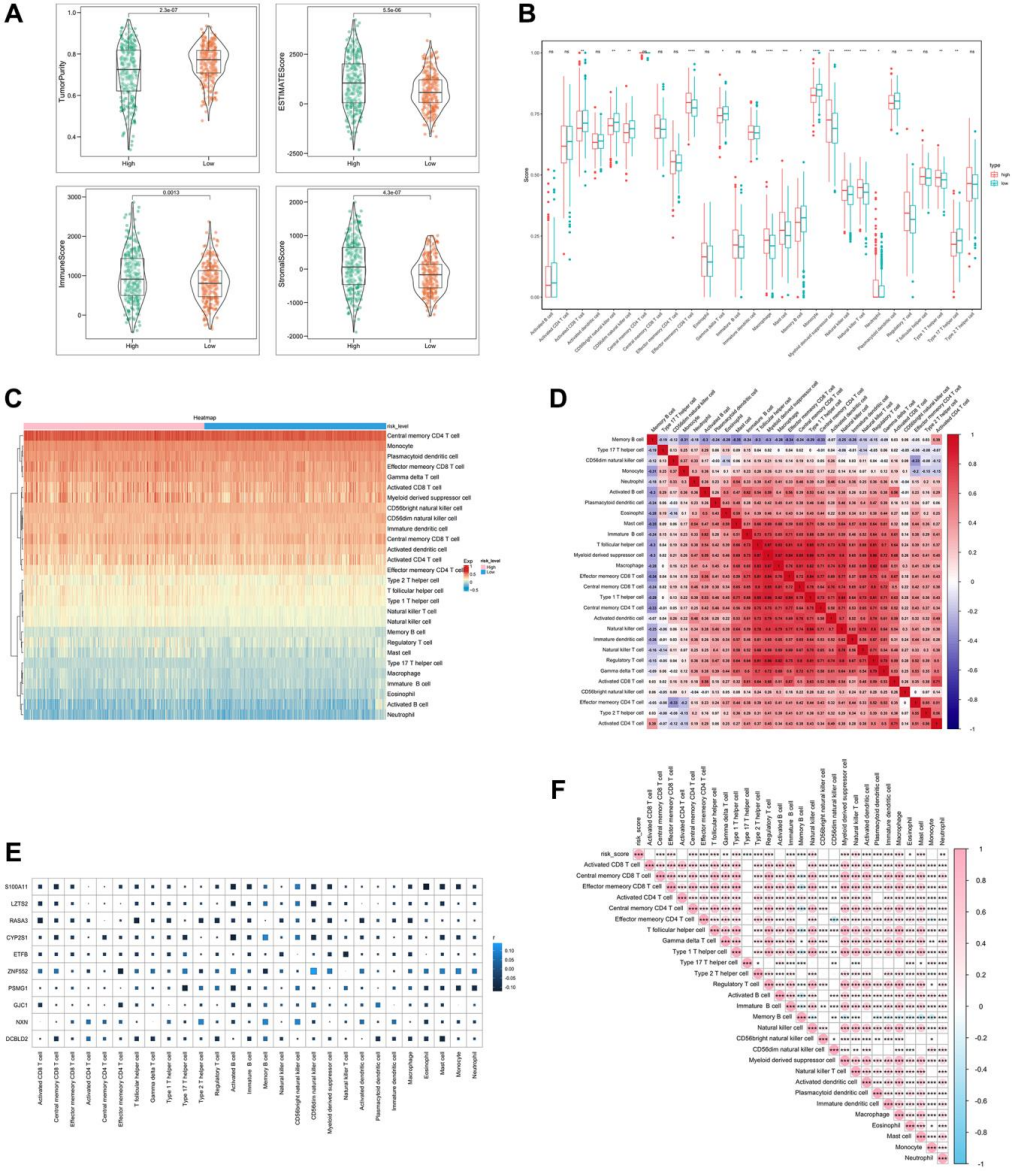
**Analysis of immune cell infiltration with SERGs risk group.** (**A**) The ESTIMATE score, Immune score, and Stromal score between high- and low- SERGs risk groups were compared. (**B**) Violin diagram of 28 type immune cells in two groups. (**C**) Immune cells assessment between two groups. (**D**) Analysis of correlation in 28 type immune cells. (**E**) Correlation analysis of ten prognostic biomarkers (S100A11, LZTS2, CYP2S1, ZNF552, PSMG1, GJC1, NXN and DCBLD2) and immune cells. (**F**) Correlation analysis of risk score and immune cells.

We investigated the correlation between SERGs risk score and immune cell infiltration by ESTIMATE algorithms and ssGSEA. SERGs high-risk group had greater ESTIMATE score, Immune score, and Stromal score levels (*P* < 0.001) (Figure 4A). ssGSEA analysis revealed that the high-risk group had significantly lower levels of activated CD8 T cells, CD56 bright natural killer cells, CD56 dim natural killer cells, gamma delta T cells, memory B cells, monocyte, Type 17 T helper cells than those in the low group. However, the levels of effector memory CD8 T cells, Macrophages, Mast cells, natural killer T cells, natural killer cells, regulatory T cells, and Type 1 T helper cells were significantly higher than those in the low group (Figure 4B). The immune cells assessment between the two groups is shown in Figure 4C. Between the 28 immune cells, macrophage was positively correlated with myeloid-derived suppressor cells (r = 0.87) and regulatory T cells (r = 0.82) (Figure 4D).

Furthermore, the correlation analysis conducted on the ten biomarkers and the immune cells indicated a strong association between ZNF552 and S100A11 with immune cells (Figure 4E). The risk score was positively correlated with regulatory T cells, immature B cells and natural killer cells, and negatively correlated with memory B cells and Type 17 T helper cells (Figure 4F). These findings demonstrate the significant difference between the two groups in the immune microenvironment and are closely associated with ZNF552 and S100A11.

### Single-cell analysis

**Figure 5:**
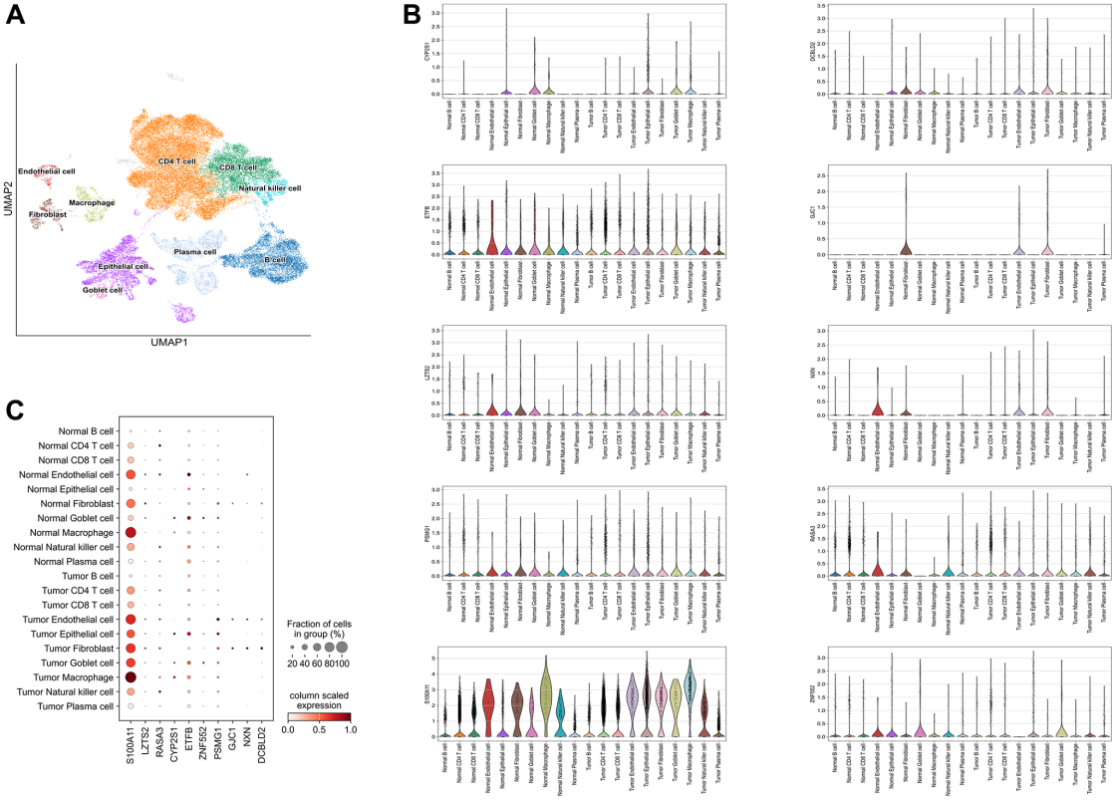
**Single-cell analysis.** (**A**) 10 types of cells were clustered. (**B**) The ten identified SERGs markers expressions were identified in CC single-cell clusters. (**C**) A bubble plot was employed to visually represent the gene expression characteristics.

The following major cell types were characterized: CD8 + T cells, CD4 + T cells, Plasma cells, epithelial cells, macrophages, B cells, Goblet cells, Natural killer cells, fibroblasts and endothelial cells (Figure 5A). S100A11 was mainly distributed in macrophages, endothelial cells, and fibroblasts. LZTS2 was mainly distributed in endothelial cells and fibroblasts (Figure 5B, 5C).

### SERGs were predictive to chemotherapy

**Figure 6:**
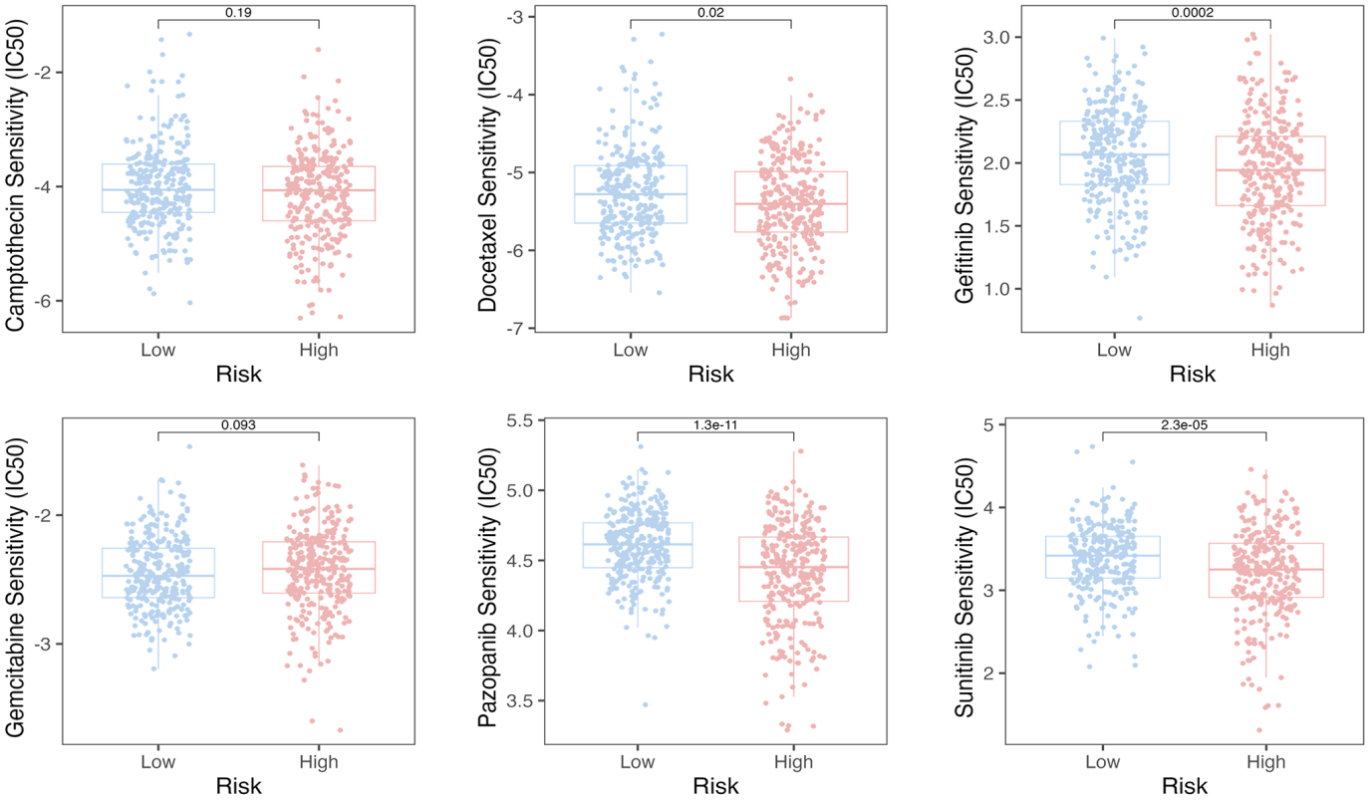
The pRRophetic algorithm predicted the IC_50_ values for six anti-cancer drugs.

We employed the pRRophetic algorithm to estimate IC_50_ values, enabling the prediction of distinct chemotherapy responses between high- and low-SERGs risk groups. Based on the GSE39582 database, we found that the high-SERGs risk group had a low IC_50_ of the anticancer drugs compared to high-SERGs risk group such as docetaxel (*P* < 0.02), gefitinib (*P* < 0.001), pazopanib (*P* < 0.001), sunitinib (*P* < 0.001), which means that the above drugs were more sensitive to the high-SERGs risk group (Figure 6).

### Identification of key gene expression in COAD

**Figure 7:**
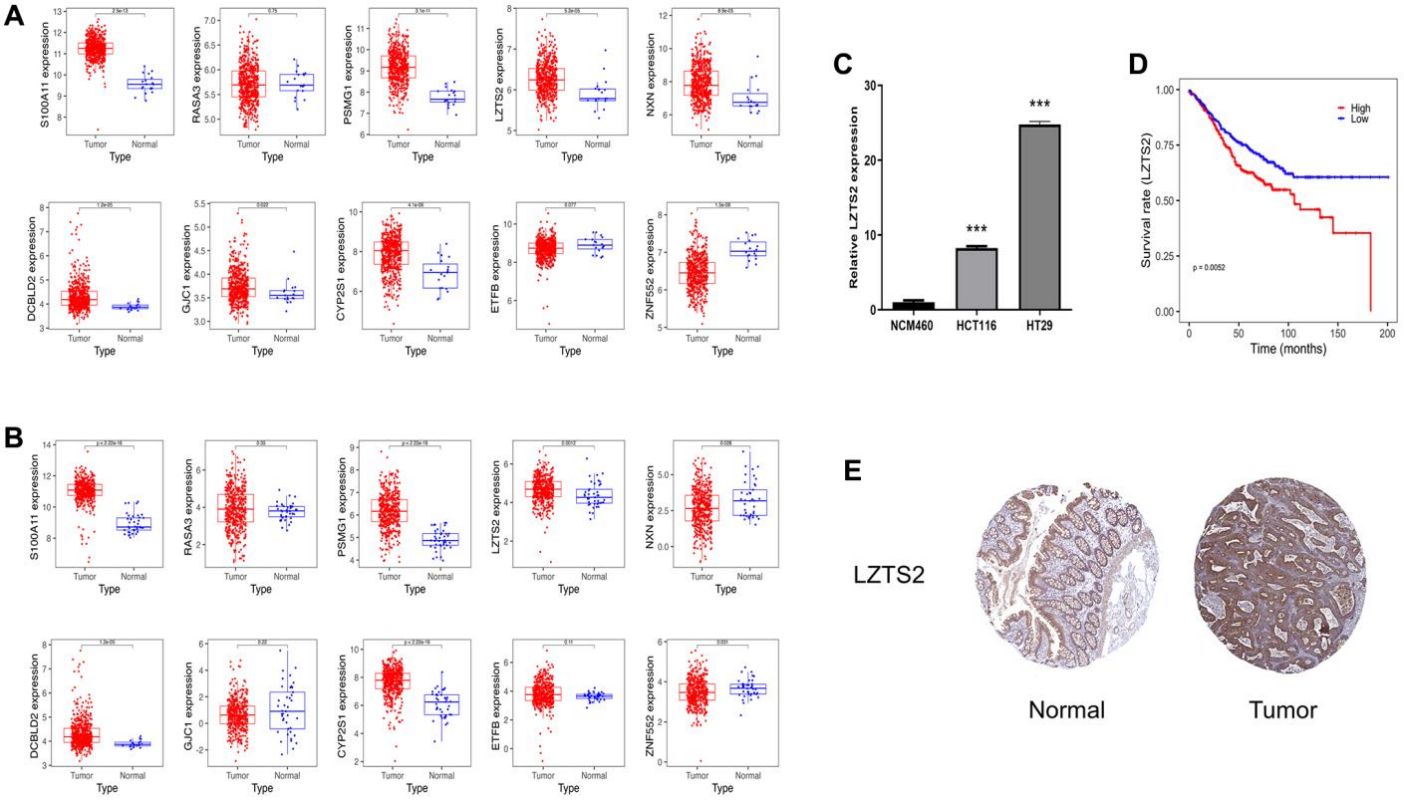
**Identification of LSZT2 mRNA expression.** (**A**) The expressions of ten genes in discovery cohorts. (**B**) The expression of ten genes in validation cohorts. (**C**) The LSZT2 expression level in NCM460 cells and CC cell lines. (**D**) The prognostic analysis of LSZT2 in CC patients. (**E**) IHC staining of LSZT2 in normal and CC tissues from the HPA database.

To further identify the key gene, we analyzed the 10 genes between cases and controls on GSE39582 and validation on the TCGA-COAD dataset. Eight genes were selected, including S100A11, LZTS2, CYP2S1, ZNF552, PSMG1, GJC1, NXN and DCBLD2 (Figure 7A). Meantime, S100A11, LZTS2, CYP2S1, PSMG1 and DCBLD2 were found on validation cohorts TCGA-COAD dataset (Figure 7B). Finally, we found that the five genes (S100A11, LZTS2, CYP2S1, PSMG1 and DCBLD2) were up-regulated in both GSE39582 and TCGA-COAD datasets with *P* < 0.05. Then, we performed qRT-PCR and found that LZTS2 was significantly up-regulated in HCT116 and HT29 cell lines compared to normal NCM460 cells (Figure 7C). The expression of S100A11, CYP2S1, PSMG1, and DCBLD2 were inconsistent with predicted results. Specifically, the five genes were queried in the GEPIA2 database, and LZST2 was selected based on “overall survival.” (Supplementary Figure 1). Notably, the WB experiment showed that the levels of LZTS2 decreased in HCT116 and HT29 cell lines than in NCM460 cells (Supplementary Figure 2). The high LZTS2 expression was related to an unfavorable OS by prognostic analysis (Figure 7D). Finally, the immunohistochemical findings retrieved from the HPA database demonstrated elevated LZTS2 protein expression within the CC tissue (Figure 7E).

## DISCUSSION

Colon cancer is the common cause of cancer-related fatalities, and its occurrence has steadily risen in recent years. The acknowledged tumor-promoting potential of SEs makes them a prospective target for immunotherapy [16, 17]. Moreover, SE has been reported to influence the development of malignancies, and SERGs can function as valuable prognostic markers [18]. Nevertheless, clinical applications remain challenging because of the absence of effective biomarkers. At the same time, few studies have explored the link between SREGs and CC. This challenge has prompted us to explore novel SERGs in CC.

In the current work, we used WGCNA to identify 8245 genes in CC, 406 of which overlapped with SEs. GO enrichment analysis revealed that BP was mainly enriched in tube morphogenesis and vasculature development, etc. CC was mainly enriched in focal adhesion, etc. MF was mainly enriched in cell adhesion molecule binding, etc. KEGG analysis was primarily related to Pathways in cancer and PI3K-Akt signaling pathway, etc. The PI3K/Akt/mTOR signaling pathway is a crucial component in the process of colorectal carcinogenesis, playing significant roles not only in the development of drug resistance but also in the initiation of metastasis in colorectal cancer [19]. Subsequently, we established and validated a ten-SERGs prognosis mode. The effectiveness of this model was further confirmed using verification datasets for the first time. The outcomes of external validation align with the previous findings, underscoring the robust predictive performance of the prognostic signature.

In this study, we noted that immune cell infiltration was notably more abundant in the high-risk SERGs group, such as memory CD8 T cells, Macrophages, Mast cells, natural killer cells, etc. The efficacy of most immunotherapies relies on the substantial infiltration of CD8+ T cells within the tumors [20–22]. Meanwhile, SE-associated lncRNAs are involved in the tumor immune microenvironment [23]. In addition, macrophages and natural killer cells play a crucial role in exerting an anti-cancer effect in tumor immunotherapy [24–27]. These findings imply that the prognostic model might serve as an indicator of immunocyte infiltration levels in two risk groups, potentially influencing patient overall survival through its impact on immunotherapy. Interestingly, high-risk CC patients were more sensitive to docetaxel, gefitinib, pazopanib, and sunitinib than low-risk patients. The combination of chemotherapy and immunity has become a trend in treating tumors. Docetaxel can potentially enhance anti-tumor efficacy by increasing the secretion of HMGB1 and CXCL11, consequently promoting the recruitment of CD8+ T cells into the tumor microenvironment [28]. Gefitinib, when combined with an immunostimulatory nanocarrier, exhibits greater efficiency in suppressing lung tumor development. This combination induces an immune-active microenvironment characterized by a higher presence of functional CD8 T cells and reduced infiltration of regulatory T cells [29]. In response, we present a promising therapeutic approach that combines conventional chemotherapy, natural products, and targeted immunotherapy directed at SERGs to enhance the infiltration of CD8+ T cells into tumors and restore sensitivity in high-risk SERGs-positive tumors to existing T-cell-based immunotherapies. Nevertheless, further research is required to develop synergistic treatment strategies.

We verified seven ten genes in the discovery and validation cohort to further identify the key gene for CC. Interestingly, our results showed that the five genes (S100A11, LZTS2, CYP2S1, PSMG1 and DCBLD2) were up-regulated in both GSE39582 and TCGA-COAD datasets with *P* < 0.05. Specifically, the five genes were queried in the GEPIA2 database, and LZST2 was selected based on “overall survival.” LZTS2 was significantly up-regulated in HCT116 and HT29 cells compared to normal NCM460 cells through PCR experiment. Notably, the WB experiment showed that the levels of LZTS2 decreased in HCT116 and HT29 cell lines than in NCM460 cells (Supplementary Figure 2). We found that the expression levels of LZTS2 mRNA were not consistent with their protein level in this study. Following the process of transcription, mRNA molecules experience a complex array of interconnected steps that ultimately lead to their translation into functional proteins. Nonetheless, the mRNA abundance of the particular gene does not always have a linear correlation with the protein expression of its translation product [30]. Gene expression is subject to a multitude of regulatory mechanisms. Both transcriptional and post-transcriptional controls, as well as translational and post-translational modulations, contribute to the ultimate expression of proteins [31, 32]. Furthermore, the levels of mRNA may not align with the levels of protein expression because of various factors, including the decay of mRNA, the breakdown of proteins, alterations in protein folding, and other regulatory influences [33, 34]. Consequently, this could account for the discrepancies between the expression levels of LZTS2 mRNA and its corresponding protein in our investigation. Moreover, it reported that the translation of LZTS2 was reduced in colorectal cancer [35]. Therefore, when it comes to disease prognosis, we need to take into account differences in both gene and protein levels. Some inhibitors targeting the translation level can also be developed in the course of clinical treatment of CC. The LZTS2 gene is on chromosome 10 at 10q24.3 [36]. It as a tumor suppressor gene, with aberrant expression implicated in the initiation and progression of certain cancers [37]. The removal of LZTS2 enhances vulnerability to tumor development [38]. LZTS2 has emerged as a novel prognostic biomarker for clear cell renal cell carcinoma and laryngeal squamous cell carcinoma [39, 40]. Nevertheless, the effects of LZTS2 in CC remain unexplored, which necessitates further investigations.

Nevertheless, this study has several limitations. Firstly, it relied on publicly available sequencing data with a relatively small sample size. Consequently, validating our prognostic model based on SERGS-related features in more extensive clinical trials is imperative. Second, cross-validation at the proteomic level is essential to ensure applicability in clinical settings. Thirdly, we lack experiments to ascertain the specific stage of CC at which LZTS2 is most effective. Utilizing the GEPIA2 database, we observed increased levels of LZTS2 in stage III CC. However, there was no significant variance in LZTS2 levels across different stages (Supplementary Figure 3). Certainly, we intend to continue our research. We plan to collect blood and tissue samples from CC patients at various stages to analyze LZTS2 expression, aiming to determine the pivotal stage in COAD development associated with LZTS2 expression. Additionally, there is currently no direct evidence demonstrating the influence of LZTS2 on prognosis via immune infiltration, and the underlying mechanisms remain unknown. Therefore, in future studies, we aim to employ flow-based techniques to elucidate the impact of LZTS2 inhibition or over-expression on immune cell distribution *in vivo*. Furthermore, we will employ immunohistochemistry to assess the distribution of immune cells in LZTS2 knockout nude mice models of CC.

## Supplementary Materials

Supplementary Figures

Supplementary Table 1

Supplementary Table 2
