# Supplementary material for: Mechanistic insights into super-enhancer-related genes as prognostic signatures in colon cancer
Source: Aging (Albany NY). 2024 Jun 7;16(11):9918–32. doi: 10.18632/aging.205906 (PMC11210223; doi:10.18632/aging.205906)
Supplement: Supplementary Figures [file aging-16-205906-s001.pdf]

SUPPLEMENTARY FIGURES

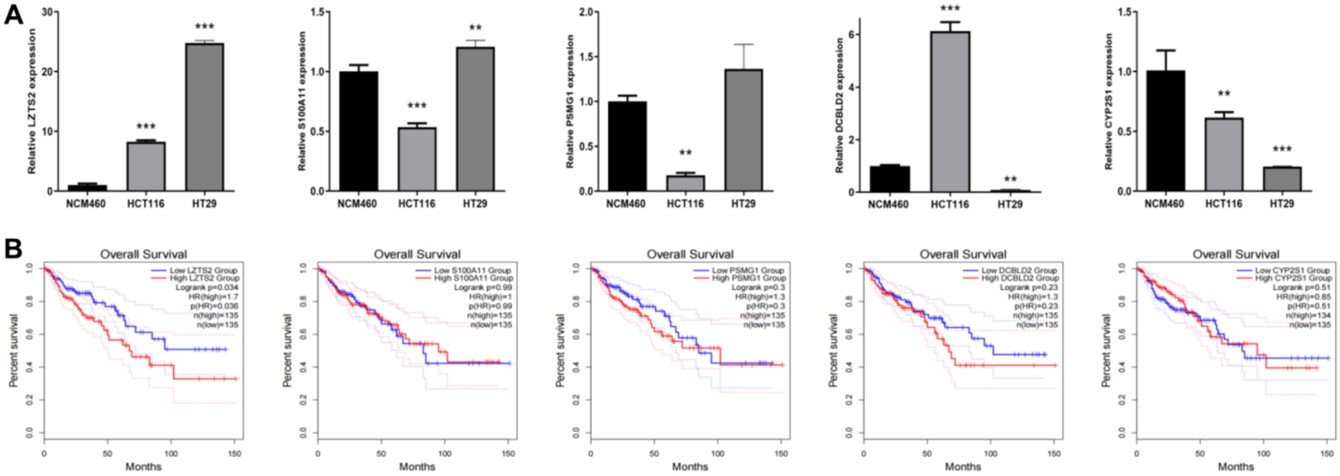

Supplementary Figure 1. Identification of key gene. (**A**) The LSZT2 expression level in NCM460 cells and CC cell lines. (**B**) The prognostic analysis of LSZT2 in CC patients.

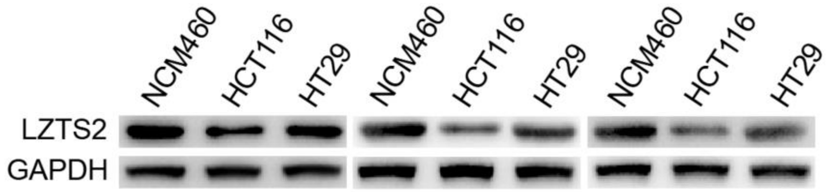

Supplementary Figure 2. The expression of LZTS2 in NCM460 cells and CC cell lines through WB analysis.

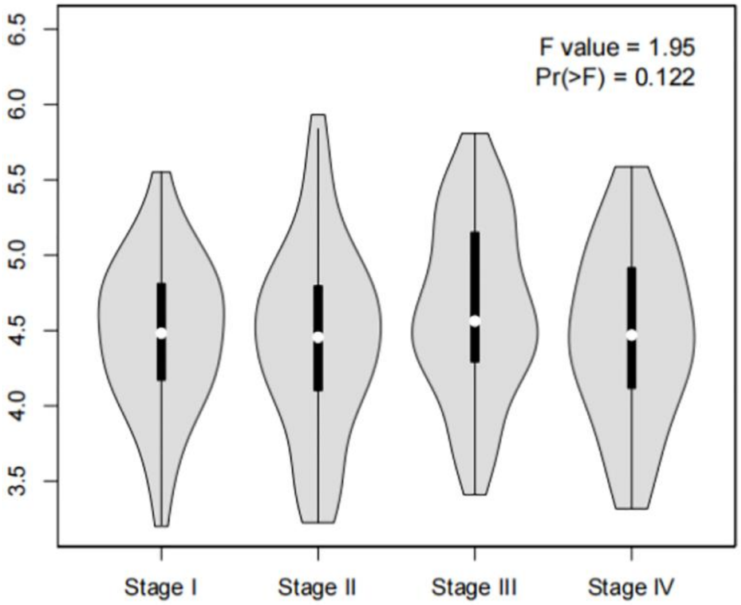

Supplementary Figure 3. The level of LZTS2 in different stages in CC through the GEPIA2 database.
