# Supplementary material for: Mechanistic insights into super-enhancer-related genes as prognostic signatures in colon cancer
Source: Aging (Albany NY). 2024 Jun 7;16(11):9918–32. doi: 10.18632/aging.205906 (PMC11210223; doi:10.18632/aging.205906)
Supplement: Supplementary Table 2 [file aging-16-205906-s003.pdf]

SUPPLEMENTARY TABLE

Supplementary Table 2. Genes with *P* < 0.01 in the univariate analysis.

	p-value	HR
S100A11	0.004343611	1.487508743
BTBD19	0.00089113	1.45870721
LZTS2	0.000522237	1.807077409
ACSL5	0.001001566	0.743193629
ITGB1	0.001205038	1.437819296
PPFIBP2	3.84E-06	0.56669651
FUT4	0.001908012	0.792675032
RASA3	0.000239138	1.953158299
HNF1B	0.002213428	0.633892564
PRR15L	1.30E-05	0.728492767
SLC25A10	0.008165605	0.696385056
GATA6	0.001019726	0.693431733
JUNB	0.006129202	1.423368894
RHPN2	8.95E-05	0.6398474
CEBPA	0.000233095	0.728040599
CYP2S1	0.006454524	0.809237313
ETFB	0.004902666	0.728385606
ZNF552	0.003001983	0.624446982
SPATA2	0.001066697	0.674213999
ETS2	0.005831722	0.783848938
PSMG1	0.009291438	0.79613124
SPRY4	0.007014512	1.475598899
ABLIM3	0.005552087	1.37781399
REPIN1	0.002972757	0.674837116
PRR15	0.001433493	0.789788149
SYBU	0.001660871	0.782693099
PLEC	0.00371291	1.522814194
ADORA2B	0.006341077	0.81708598
GJC1	0.006357807	1.726527912
NXN	0.009991729	1.193947245
GADD45B	0.000771276	1.344235954
WWTR1	0.004248447	1.349251887
DCBLD2	7.00E-07	1.614855247
ANGPT2	0.001023235	1.311722989
